# Eradication of *Salmonella *Typhimurium infection in a murine model of typhoid fever with the combination of probiotic *Lactobacillus fermentum *ME-3 and ofloxacin

**DOI:** 10.1186/1471-2180-8-132

**Published:** 2008-08-04

**Authors:** Kai Truusalu, Raik-Hiio Mikelsaar, Paul Naaber, Tõnis Karki, Tiiu Kullisaar, Mihkel Zilmer, Marika Mikelsaar

**Affiliations:** 1 Department of Microbiology, University of Tartu, Estonia; 2 Department of Biochemistry, University of Tartu, Estonia; 3 Department of Medical Microbiology, Stavanger University Hospital, Norway

## Abstract

**Background:**

The aim of the study was to detect whether in experimental *Salmonella enterica *Typhimurium infection the probiotic *Lactobacillus fermentum *ME-3 in combination with fluoroquinolone therapy would eradicate *S. *Typhimurium, prevent the development of liver and spleen granulomas and improve the indices of oxidative stress in the ileum mucosa.

The selected bacteriological, histological and biochemical methods were applied.

**Results:**

Combined treatment with *L. fermentum *ME-3 and ofloxacin eradicated *Salmonella *Typhimurium from blood, ileum and liver, decreased the number of animals with liver and spleen granulomas and reduced the value of lipid peroxides in the ileum mucosa. Higher total counts of intestinal lactobacilli in all experimental groups were associated with the absence of liver granulomas.

**Conclusion:**

The antimicrobial and antioxidative probiotic *L. fermentum *ME-3 combined with ofloxacin enhances the eradication of experimental *S*. Typhimurium infection. These observations on probiotic and antimicrobial co-action may serve as basis to develop new strategies for treatment of invasive bacterial infections of the gut.

## Background

Typhoid fever is a systemic infection caused by *Salmonella enterica *serotype Typhi with 22 million of new cases registered annually worldwide despite various vaccination programs [1,2]. The prevalence of hepatobiliar system involvement is considered in 1–26% of patients with typhoid fever [3].

Broad-spectrum fluoroquinolones are the drugs of choice for the treatment of typhoid fever [2] inhibiting even fastidious intracellular pathogens in therapeutic concentrations readily achievable in body fluids and tissues [4]. Despite the antimicrobial treatment almost 1–6% of infected patients become chronic carriers [1] shedding bacteria in their stools and urine for a varying period of time.

One possibility to devise new strategies in treatment of bacterial gastrointestinal infections seems to be the application of probiotics as an adjunct to chemotherapy. Probiotic strains are defined as live microorganisms which, when consumed in appropriate amounts in the food, confer a health benefit on the host [5]. A number of clinical trials with controversial results have been performed with probiotics in the prevention and treatment of gastrointestinal infections, caused by rotavirus and *Clostridium difficile *[6-8]. In the management of *Helicobacter pylori *infection probiotics in combination with antibiotic treatment have been reported to be successful [9-11].

We applied a new probiotic *Lactobacillus fermentum *ME-3, DSM 14241 (ME-3) [12] with high antimicrobial activity, particularly against gram negative pathogens [12,13]. Besides, ME-3 has considerable resistance to several reactive oxygen species (ROS) and possesses substantial antioxidative activity demonstrated by expression of manganese superoxide dismutase [14].

For experimental investigation of *Salmonella *Typhi infection a mouse model is reliable and it has been successfully applied also in earlier studies [15,16]. The infection due to *S*. *enterica *serotype Typhimurium in mice resembles human typhoid fever with extra-intestinal granulomatous lesions in different organs [2,17,18].

The aim of the current study was to detect whether in experimental *S. *Typhimurium infection the probiotic ME-3 in combination with fluoroquinolone therapy would eradicate *S*. Typhimurium from host, prevent the development of liver and spleen granulomas and improve the indices of oxidative stress in the ileum mucosa.

## Results

### *In vitro *tests

The MIC values of ofloxacin (OFX) to *S. *Typhimurium were 0.19 μg/ml and to ME-3 8 μg/ml. When the *S. *Typhimurium and ME-3 were tested together a six-fold decrease in the MIC of OFX from 0.19 to 0.032 μg/ml was observed with both applied methods (overlay and broth dilution).

### Experimental infection

All but two animals from *S. *Typhimurium group (Gr1) survived up to the end of the experiment. The survival rate in Gr1 was 91% and in other groups 100% on Day 10. As demonstrated in Table 1 by Day 10 in Gr1 mice, challenged with *S. *Typhimurium, the viable bacteria were isolated from the ileum, blood and the liver (11/20, 3/20 and 11/20, respectively). The administration of OFX (Gr2) and ME-3 (Gr3) significantly reduced the number of mice with viable *S. *Typhimurium in the ileum (p = 0.0032). Besides, *S*. Typhimurium was still cultivated from blood and liver of some animals of Gr2 and Gr3. While, viable *S. *Typhimurium were not found in any of investigated sites applying the combination of OFX and ME-3 (Gr4).

**Table 1:** The number of mice with viable *S*. Typhimurium and granulomas in liver and spleen

Experimental groups	Number (%) of mice with				
	
	*Salmonella *Typhimurium			Granulomas	
	
	ileum	blood	liver	liver	spleen
Gr1 (ST)	11^1;2^	3	11^3^	16^4;5^	11^7^
n = 20	55%	15%	55%	80%	55%

Gr2 (ST+OFX)	2^1^	1	3	8^6^	0^7;8^
n = 13	15%	8%	23%	62%	

Gr3 (ST+ME-3)	2^1^	0	1^3^	5^4^	4^8^
n = 13	15%		8%	38%	31%

Gr4 (ST+OFX+ME-3)	0^2^	0	0	2^5;6^	0^7;8^
n = 13				15%	

ST – *Salmonella *Typhimurium

OFX – ofloxacin

ME-3 – *Lactobacillus fermentum *ME-3

^1 ^p = 0.032 Gr1 *vs *Gr2 and Gr3 viable ST in ileum

^2 ^p = 0.002 Gr1 *vs *Gr4 viable ST in ileum

^3 ^p = 0.009 Gr1 vs Gr3 viable ST in liver

^4 ^p = 0.027 Gr1 *vs *Gr3 liver granulomas

^5 ^p < 0.001 Gr1 *vs *Gr4 liver granulomas

^6 ^p = 0.023 Gr2 *vs *Gr4 liver granulomas

^7 ^p = 0.002 Gr1 *vs *Gr2 and Gr4 spleen granulomas

^8 ^p = 0.048 Gr2 *vs *Gr3 and Gr4 *vs *Gr3 spleen granulomas

The frequent histopathological findings were the granulomas in liver and spleen, detected in more than half of the untreated animals (Gr1). In the spleen (Table 1) the treatment with OFX as single and in combination with ME-3 were superior to treatment by ME-3 alone (both p = 0.048). The number of mice with liver granulomas was significantly reduced by application of the OFX and ME-3 combination as compared to OFX alone (15% *vs *62%, p = 0.023).

Lactobacilli were present in terminal ileum of all investigated animals (median counts 8.6 log_10 _CFU/g). Numerous colonies of *L. fermentum *as species were detected in animals of Gr3 and Gr4. However, we could not distinguish the presence of the strain *L. fermentum *ME-3. A somewhat increased count and smaller variation of lactobacilli was detected in groups of animals treated with *L. fermentum *ME-3 (Gr3 and Gr4 mice: range 8.7–9.6 median 9.1 *vs *Gr 1 and 2: 6.8–8.8 median 7.7). No translocation of lactobacilli into blood and liver was detected. In all experimental groups the higher total counts of intestinal lactobacilli were associated with the absence of granulomas in the liver (p = 0.002).

The indices of oxidative stress *e.g*. the level of LPO and GSSG/GSH were higher in mice challenged with *S. *Typhimurium as compared to control group (p < 0.001 and p < 0.003 respectively) (Table 2). The LPO values were reduced significantly (p = 0.002) in both treatments: with ME-3 alone (Gr3) and in combination with OFX (Gr4) as compared with that of OFX (Gr2).

**Table 2:** Indices of oxidative stress (with standard deviations) in the ileum mucosa in mice challenged with *S. *Typhimurium and treated with ofloxacin and/or the probiotic *L. fermentum *ME-3

Experimental groups	LPO (pmol/mg protein)	GSSG/GSH
Gr1 (ST)	338 ± 46^1;4^	0.26 ± 0.41^3;5^
Gr2 (ST+OFX)	228 ± 41^2^	0.26 ± 0.11
Gr3 (ST+ME-3)	169 ± 11^1;2^	0.16 ± 0.20^3^
Gr4 (ST+OFX+ME-3)	161 ± 27^1;2^	0.17 ± 0.11^3^
Control (PBS)	157 ± 24^4^	0.11 ± 0.2^5^

ST – *Salmonella *Typhimurium

OFX – ofloxacin

ME-3 – *Lactobacillus fermentum *ME-3

PBS – phosphate buffered saline

LPO – lipid peroxides

GSSG/GSH – glutathione redox ratio

^1^p < 0.001 Gr1 *vs *Gr3 and Gr4

^2^p = 0.002 Gr2 *vs *Gr3 and Gr 4

^3 ^p = 0.006 Gr1 *vs *Gr3 and Gr4

^4^p < 0.001 Gr1 *vs *Control

^5^p < 0.003 Gr1 *vs *Control

## Discussion

We have previously shown that ME-3 has antagonistic activity against *S. enterica *serovar Typhimurium as well as *in vitro *[13] and *in vivo *mice model [16].

In this experimental study we explored the influence of combined per oral treatment with OFX and *L. fermentum *ME-3 to *S*. Typhimurium infection. The infection mimics human typhoid fever characterised by specific granulomatous lesions in various organs [3]. In the current study the granulomas were detected in liver and spleen of S. Typhimurium challenged mice.

Although according to *in vitro *tests *S. *Typhimurium strain was susceptible to OFX, the treatment did not eradicate *S. *Typhimurium from ileum, blood and liver. By contrast, OFX combined with probiotic ME-3 completely eradicated viable *S. *Typhimurium from investigated sites and prevented the formation of spleen granulomas, at the same time significantly reducing the presence of liver granulomas. To the best of our knowledge, this is the first time that a *Lactobacillus *probiotic in combination with a fluoroquinolone has been shown to exert beneficial effect on the treatment of infectious granulomatous inflammation.

We observed an interesting difference between spleen and liver in the prevention of the granulomas during combinative therapy of *S. *Typhimurium infection: the number of mice with liver granulomas was higher than the corresponding data of spleen. Obviously, this is due to the more expressed immune-competence of spleen as compared to liver.

The limitation of our study was that we did not differentiate between the *L. fermentum *ME-3 strain and any indigenous *Lactobacillus *strains present in the mice gut during the experiment. However, under the combinative treatment with *L. fermentum *ME-3 and ofloxacin besides the eradication of viable *S. *Typhimurium the significant reduction of granulomas in liver and their total absence in spleen was found.

Concerning the application of probiotics in gastrointestinal infections, the exact mechanism of action is not known yet. Four possible mechanisms can be suggested. First, it has been shown that lactobacilli produce several substances like antimicrobials, lactic and non-lactic acids, hydrogen peroxide which enables to inhibit or kill pathogens [19]. Second, in the gut lactobacilli seemingly compete with the pathogen for the adhesion sites and nutritional sources [6]. Third, lactobacilli usually cause the immune-modulation of the host increasing the resistance against pathogens and fourth, they are able to inhibit the production of bacterial toxins [20]. All abovementioned factors seemingly might be involved in the eradication of *Salmonella *Typhimurium infection.

In the mice model the *L. fermentum *ME-3 apparently suppressed *Salmonella *Typhimurium due to previously established high antimicrobial activity and production of both lactic and acetic acids and generation of H_2_O_2 _[13]. This was in accordance with the current *in vitro *tests, where MIC values of OFX to *S. *Typhimurium in combination with ME-3 decreased even six-fold.

Remarkably, in mice with high numbers of intestinal lactobacilli the granulomas of liver and spleen were not found. Probably the increased counts of total lactobacilli inhibited the spread of *S. *Typhimurium into organs and prevented the formation of granulomas. This could have been associated with hyperplasia of lymph nodes in ileum, found previously after administration of ME-3 [16] and involved also in the present study. (data not shown)

The absence of translocation of *S. *Typhimurium and intestinal lactobacilli as much as the reduced invasion of *S. *Typhimurium into tested organs could be explained by improved anti-oxidative status of gut mucosa. During the intracellular infection phagocytes produce reactive oxygen species (ROS), important for killing the pathogen. Though the formation of ROS is reported to be induced also by fluoroquinolones, their role in the antibacterial action is not clearly understood [21]. The excessive amounts of ROS damage the collateral intestinal epithelial cells and the superoxide compound was responsible for the generation of the granulomatous lesions limiting the spread of infection [22]. In the current study it was shown that the administration of this antioxidative probiotic to infected mice significantly reduced LPO values of ileum mucosa in comparison with the gut of the untreated and treated with OFX animals. Thus, the application of *L. fermentum *ME-3 suppressed the excessive oxidative stress indices and could have improved the oxidative state of the gut mucosal tissue. Apparently, the neutralisation of the produced superoxides by superoxide dismutase of ME-3 was one of the putative mechanisms in prevention of the granulomatous lesions [14]. Recently, the concept of oxidative stress has been advanced as "a disruption of redox signalling and control" [23]. This emphasises the impact of glutathione and its redox ratio in intestinal cells during *S*. Typhimurium infection [16]. In the current study the addition of ME-3 to OFX reduced glutathione redox ratio by improving the oxidative status in ileum.

## Conclusion

We conclude that the antimicrobial and antioxidative probiotic *L. fermentum *ME-3 combined with ofloxacin enhances the eradication of experimental *S*. Typhimurium infection. These observations on probiotic and antimicrobial co-action could serve as basis to develop new strategies for treatment of invasive bacterial infections of the gut.

## Methods

### Bacterial strains and susceptibility testing

A clinical isolate of *Salmonella enterica *serovar Typhimurium was kindly provided by the Estonian Laboratory of Public Health Inspectorate.

For treatment of the experimental *S*. Typhimurium infection a fluoroquinolone ofloxacin (Hoechst, Germany) and a probiotic *L. fermentum *ME-3 were applied. The MIC values of ofloxacin to *S. *Typhimurium on Mueller-Hinton media (Oxoid, UK) were measured by the E-test following the manufacturer's instructions (AB Biodisk, Sweden) and estimated according to the CLSI guidelines [24]. *Lactobacillus fermentum *ME-3 originated from the Microbial strains collection of Department of Microbiology of University Tartu.

The following two tests were used to evaluate the combinative effect of OFX and ME-3 against *S. *Typhimurium. First, in the overlay test, 10 ml of the MRS agar (Oxoid, UK), containing 10^8 ^CFU/ml of ME-3, was poured onto agar plates and incubated in 10% CO_2 _at 37°C for 48 h. Then the plates were overlaid with 5 ml 1.0% (w/w) Isosensitest agar (Oxoid, UK) and 10^8 ^CFU/ml of *S. *Typhimurium was seeded into agar. Plates were incubated in microaerobic conditions at 37°C for 24 h and E-test was applied. Second, in the dilution test, serial two-fold dilutions of OFX in broth were prepared. *S. *Typhimurium and ME-3 solutions were adjusted to the 0.5 McFarland turbidity standards and 10 μl of the suspension was placed into the OFX broth (Nutrient broth No2 Oxoid, UK) and the MBC values were detected. All susceptibility tests were performed in duplicate.

### Experimental murine model

The 4 to 6 week old NIH line mice (Kuopio, Finland) were inoculated orally by a single 0.5 ml dose of the *S. *Typhimurium suspension (10^5 ^CFU/ml) using a sterile syringe with the blunt-ended tube. After 48 hours animals were treated either with OFX or ME-3 alone or with their combination for 8 days. Control animals received PBS (phosphate buffered saline). OFX at doses of 20 mg/kg [25] was diluted in 0.5 ml of PBS and given by the sterile syringe with the blunt-ended tube once daily.

Lyophilised ME-3 (Probiotical s.r.l, Novara, Italy) was suspended in PBS to a final concentration of 5 × 10^7^CFU/ml. During the experiments each mouse consumed daily approximately 5 millilitres of ME-3 containing PBS, receiving 2.5 × 10^8 ^CFU of lactobacilli.

The commercial diet R-70 (Lactamin, Sweden) with tap water was given *ad libitum*.

A total of 61 mice were infected with *S. *Typhimurium and divided into following groups: Gr1 (untreated; n = 22), Gr2 (treated with OFX; n = 13), Gr3 (treated with ME-3; n = 13) and Gr 4 (treated with OFX and ME-3; n = 13). In addition, 11 uninfected animals were treated with PBS and served as a control group for the biochemistry testing. On Day 10 all the surviving animals were sacrificed by cervical dislocation and an autopsy was performed. All experiments were approved by the Committee of Animal Experiments of Estonian Ministry of Agriculture.

### Bacteriological assays

At autopsy 10 μl of the heart blood was cultured onto McConkey agar (Oxoid, UK) and on the de Man-Rogosa-Sharpe (MRS) medium (Oxoid, UK) for detection of *S. *Typhimurium and *L. fermentum*, respectively. The samples of the ileum and liver were homogenized with sterile glass powder and 10 μl of homogenate was cultured onto McConkey agar. All plates were incubated in the air at 37°C for 24 h. For quantification of lactobacilli the homogenised samples from liver and ileum were weighed and 10 μl of serial ten-fold dilutions in PBS (pH 7.2) were cultured on the MRS and incubated in the 10% CO_2 _at 37°C for 48 h. The total counts of lactobacilli were calculated as CFU/mg. The lower limit of detection was ≥ 3.0 log CFU/ml. For identification of *Lactobacillus fermentum *ME-3 the following criteria were applied: colony morphology on MRS, negative catalase reaction, growth on 15°C, lysozyme production and gas production from glucose [26].

### Histology

Samples from the ileum, liver and spleen were fixed in 10% formaldehyde and processed further for paraffin embedding. Tissue sections were stained by haematoxylin and eosin. Two pathologists independently in a blinded manner using coded slides evaluated inflammatory and/or destructive lesions. The focal collections of inflammatory cells with various degrees of necrosis were defined as granulomas [27].

### Biochemistry

The ileum mucosa collected at autopsy was stored at -80°C until further analysis for a maximum of three months. The indices of oxidative stress: LPO (lipid peroxides) and GSSG/GSH (glutathione redox ratio) were measured after homogenization in a 1.15% KCL solution (1:10).

The levels of LPO were detected using the commercial kit Bioxytech LPO-586 (Oxis International, USA). The value of the redox status of the ileum mucosa was expressed as the glutathione redox ratio GSSG/GSH. The total glutathione TSSG and the oxidized glutathione GSSG of the ileum mucosa were measured using the Griffith method [16,28]. Glutathione content was determined on the basis of a standard curve generated with a known concentration of this substance. The amount of reduced glutathione GSH was calculated as the difference between TSSG and GSSG.

### Statistics

The computer program Sigma Stat for Windows 2.0 (Jandel Corporation, USA) was applied. The tests were selected according to data distribution: the Fisher exact test in comparing categorical values, the Student t-test with Bonferroni correction for describing the continuous indices. The Mann-Whitney test was used for comparing unevenly distributed data.

## Competing interests

*Lactobacillus fermentum *ME-3 and/or its use as a probiotic has been patented (Estonian patent EE4580, US patent US7244424, Russian Federation patent RU2284354, European patent application EP1401457, international patent application WO03002131). The owner of the patents is the University of Tartu. The authors are being rewarded proportionally according to their contribution towards the creation of intellectual property in accordance with the Order of Handling Inventions valid at the University of Tartu.

## Authors' contributions

KT, PN and MM designed the experiments. KT performed microbiological studies and wrote the manuscript, TKa performed statistical analysis and RHM performed histological studies. TKu and MZ were responsible for biochemical experiments. PN, R–HM, MM and MZ participated in the writing of the manuscript. All authors read and approved the final manuscript.
